# Chemical Examination of Baking Powders

**Published:** 1880-09

**Authors:** R. S. G. Paton

**Affiliations:** Chemist of Health Departments


### ARTICLE V.

LABORATORY OF THE HEALTH DEPARTMENT,
CHICAGO, August 3, 1880.

OSCAR C. DEWOLF, A. M., M. D.,
*Commissioner of Health*,

DEAR SIR:—I have made a very careful and elaborate chemical examination of twenty-seven samples of baking powders received from you through the hands of Mr. Merki, your clerk, and have the honor to report as follows:

Many manufacturers have expended large sums of money and a great deal of time, attention, etc., to make up what they term a "good formula." It would not be fair to them, nor do I think it the place of this department to publish a complete analysis of of each and every baking powder. What is necessary is simply to point out, are there any, and if so, what materials contained therein deleterious to the health of the community?

I here place a table of figures and shall explain them below:

| No. | Name. | Soluble. Ash. | Insoluble Ash. | Total Ash. | |
|---|---|---|---|---|---|
| 1. | Silver | 22.00 | 0.02 | 22.02 | Ca. |
| 2. | Perfection | 39.00 | 0.06 | 39.06 | 1st. |
| 3. | American | 24.06 | 0.10 | 24.16 | Ca. |
| 4. | Chartres | 18.52 | 0.02 | 18.54 | Al. |
| 5. | Standard, Thompson & Taylor | 29.44 | 0.30 | 29.74 | 1st. |
| 6. | Gillett's Yeast | 30.68 | 0.12 | 30.80 | Ca. |
| 7. | Gillett's Cream | 31.74 | 0.08 | 31.82 | 1st. |
| 8. | Dr. Price's Cream | 36.18 | 0.12 | 36.30 | 1st. |
| 9. | Loyal | 18.56 | 1.04 | 19.60 | 1st. |
| 10. | Anchor | 30.78 | 1.28 | 32.06 | Al. |
| 11. | Standard, St. Bernard Mills | 31.52 | 0.48 | 32.00 | Ca. |
| 12. | Sterling | 29.60 | 0.20 | 29.80 | 1st. |
| 13. | Sherman's Pure | 33.84 | 0.08 | 33.92 | 1st. |
| 14. | Empress | 22.70 | 0.60 | 23.30 | Al. |
| 15. | Union Mills | 40.54 | 0.16 | 40.70 | 1st. |
| 16. | Cottage | 35.86 | 0.08 | 35.94 | 1st. |
| 17. | Royal | 36.10 | 0.12 | 36.22 | 1st. |
| 18. | Reliable | 27.00 | 0.60 | 27.60 | Al. |
| 19. | Chapman's | 18.92 | 0.24 | 19.16 | Al. |
| 20. | Giant | 20.06 | 0.34 | 20.40 | Al. |
| 21. | Manuf'd in bulk, by Union Mills | 22.62 | 0.66 | 23.28 | Al. |
| 22. | Meeches' | 22.08 | 0.28 | 22.36 | Ca. |
| 23. | Success | 26.28 | 0.12 | 26.40 | Al. |
| 24. | Golden Harp | 19.20 | 1.70 | 20.90 | 1st. |
| 25. | Star Chemical Works | 35.02 | 2.32 | 37.34 | 1st. |
| 26. | Peerless | 19.96 | 2.40 | 22.36 | 1st. |
| 27. | Manuf'd in bulk, by Star Chemical Works | 26.92 | 0.24 | 27.16 | Al. |

A known and weighed quantity of baking powder was incinerated in a silver crucible until only a gray residue was left; this is the total ash. It was carefully removed to a glass vessel and acted upon by by hydrochloric acid and distilled water at the boiling point; what dissolved in that menstruum has been called soluble ash; that which did not, insoluble ash. From the above table it will be noticed that the insoluble ash is comparatively small excepting in Nos. 9, 10, 24, 25 and 26. It may fairly be assumed that that amount of the baking powder will not be dissolved by the juices of the stomach and may therefore be quoted as objectionable in these samples.

There are really four kinds of baking powder in the market.

1st. One composed of cream of tartar and bicarbonate of soda, or, what is essentially the same, of cream of tartar, bicarbonate of soda with some wheat or rice starch. The starch is added in order to keep the other ingredients apart and prevent their reacting upon one another until required to do so. The result of kneading such powders with flour and water is the production of carbonic acid gas and a nice light porous bread. In baking in the oven a second portion of carbonic acid gas may be given off from the decomposition of the tartaric acid of the cream of tartar. Those marked first (1st) on the above table belong to this class.

2nd. One manufactured from meal and pressed yeast dried together. This would of necessity make an excellent baking powder were it not that it does not keep well for any length of time especially in warm weather. None of those samples I have received from you have been made of such materials.

3d. Another class is made from the acid phosphate of calcium and bicarbonate of soda with flour or some kind of starch. It is well known that the central portion of the kernel of all cereals contains almost pure starch; that is to say that it has almost no nitrogenous matter, moisture nor ash with it. The external part, that immediately under the husk—is, however, rich in nitrogen and phosphates. The former (nitrogen) is the characteristic ingredient of the gluten which imparts the yellow to brown tint of flour. That color some consider objectionable. The phosphates are the bone producing constituents of the flour and this class of baking powders proposes to rectify the insalubrity of the pure white or highest grade flours. It is necessary to keep in mind that no one can live on a purely starch diet alone but that in order to form all the various tissues of the body a variety of substances is required. This is very clearly demonstrated by the fact that unbolted wheat is very much more nutritious than the finest white flour. Under the next class I shall again refer to this. The baking powders of this—the third-class serve the purposes for which they are intended remarkably well. The acid calcic phosphate itself is soluble in water; any changes which may occur to it in the process of firing and kneading will not render it insoluble in the gastric juice; it will therefore in time fulfill all that is required of it.*

4th. The next class of baking powders is that in which the ingredients are alum, and bicarbonate of soda, or alum, bicarbonate of soda and starch or flour of some kind. The addition of

---

* Those marked Ca. on the table belong to the 3rd-class.

starch in this case is for the same reason as in the others and cannot be objected to unless where an excessive amount is used. With regard to alum baking powders there seems to be a great variety of opinion. Why there should be I cannot see. The addition of alum to flour in the presence of water will render the albuminous (flesh forming) constituents thereof insoluble. Does not that as well deteriorate the value of the flour as cause the stomach and intestines an amount of labor to turn over and get rid of useless material? Yet you will say, "Do not these albuminous substances cause the yellow tint of the flour and bread?" Certainly they do, as I have mentioned under 3rd class, yet their usefulness vastly overbalances their objectionableness in color. I cannot do better than incorporate here the opinion of Dr. Hassall of London on the use of alum in bread as I entirely concur in all he says on this subject, "The use of *alum* in bread is *particularly injurious.* It is true that it causes the bread to be whiter than it would be otherwise; indeed whiter than it was ever intended to be by nature; but it imparts to bread several other properties: thus it hardens the nutritious constituent of the bread, the gluten, and so, on the authority of the great chemist, Liebig, renders the bread more indigestible; it enables the baker to adulturate his bread with greater quantities of rice and potatoes than he could otherwise employ; and lastly, by the use of alum he is able to pass off an inferior, and even a damaged flour, for one of superior quality. Is it worth while to injure the properties of the bread by using alum for the sake of obtaining an unnaturally white loaf? The public then, in judging of the quality of the bread by its color—by its whiteness—commits a most serious mistake; there is little or no connection between color and quality; in fact, very generally, the whitest breads are the most adulturated. The public, therefore, should lose no time in correcting its judgment on this point.

The outer part of the grain of wheat has been proven by analysis to be much richer in nourishing principles, in gluten and in oily matter especially, than the central and more floury parts of the grain. Now, in preparing the finer descriptions of flour, the utmost pains are taken to separate this highly nutritious exterior portion of the grain, and thus, although the flour so obtained is very fine and white—very suitable for making a white loaf, that fallacious test of quality—it is yet not nearly so nutritious as whole meal flour, or even the less finely dressed qualities of wheat flour. The consumer, now better instructed, is in a position to judge of how much he sacrifices for the mere sake of an arbritary and fallacious standard of quality, namely whiteness. The difference in nourishing properties between whole meal flour and finely dressed flour amounts in many cases to fully one-third.

Further, alum is very apt to disorder the stomach, and to occasion acidity and dyspepsia. The manner in which it does so has not been clearly ascertained. The powerful effects of alum as an astringent when administered as a medicine, are well known; but when added to flour or bread, it becomes decomposed, sulphate of potash, an aperient salt being formed.

Liebig considers that part of the beneficial action of wheat flour on the system is due to the soluble phosphates which it contains in such large quantities, and he states that when alum is added to bread these are decomposed, the phosphoric acid of the phosphates uniting with the alumina of the alum, and that thus an insoluble phosphate of alumina is formed, and the beneficial action of the phosphates consequently lost to the system. So satisfied is Liebig that this is the case, that he has recommended the employment of small quantities of lime water for the purpose of whitening bread made from musty or damaged flour. Enough has now been adduced to show that it is a very dangerous thing to tamper with articles of daily food and of large consumption, like flour and bread, by the addition of chemical substances of any kind."—*Hassall, food and its adulterations.*

I have carefully examined these samples for the presence of highly dangerous or poisonous metals and find them all to be perfectly free therefrom. The only objectionable metal to be found in them is alum as under class 4. These have been distinguished in the above table by the letters Al.

Upon the whole, I believe in the use of baking powders, as manufacturers on the large scale can more readily secure pure ingredients and proper proportions than can private individuals simply making a small quantity for their own use, or using the various ingredients seperately.

I should, on no account, however, permit the use of baking powders in which alum is one of the chief constituents.

Some of the above powders contain alum in small quantities but as a complete analysis has not been given it is not mentioned in the table. I have the honor to be, sir,

Yours very respectfully,

R. S. G. PATON,

*Chemist of Health Department.*

